# Applicability of Different Methods for Quantifying Virucidal Efficacy Using MENNO Florades and Tomato Brown Rugose Fruit Virus as an Example

**DOI:** 10.3390/plants12040894

**Published:** 2023-02-16

**Authors:** Shaheen Nourinejhad Zarghani, Jens Ehlers, Mehran Monavari, Susanne von Bargen, Joachim Hamacher, Carmen Büttner, Martina Bandte

**Affiliations:** 1 Division Phytomedicine, Faculty of Life Sciences, Albrecht Daniel Thaer-Institute of Agricultural and Horticultural Sciences, Humboldt-Universität zu Berlin, Lentzeallee 55-57, 14197 Berlin, Germany; 2 Section S.3 eScience, Federal Institute for Materials Research and Testing, Unter den Eichen 87, 12205 Berlin, Germany; 3 INRES—Plant Pathology, Universität Bonn, Nussallee 9, 53115 Bonn, Germany

**Keywords:** RT-qPCR, qELISA, bioassay, local lesion, *Nicotiana* spp., TaqMan, disinfection

## Abstract

After entry of a quarantine/regulated pathogen, infected plants shall be destroyed, and the cultivated area (e.g., greenhouse) shall be disinfected. Therefore, the selection of an effective disinfectant plays an important role. With the availability of different methods for virus quantification, we investigated the application of quantitative ELISA (qELISA), RT-qPCR (reverse transcription-quantitative polymerase chain reaction), and bioassays for the quantification of disinfectant efficacy. Therefore, we estimated the titer reduction in tomato brown rugose fruit virus (ToBRFV), a regulated pathogen, in plant sap and on germ carriers after treatment with MENNO Florades 4% for 16 h. The virus load before and after the treatment was measured with the mentioned methods. The RT-qPCR and qELISA methods showed very low efficacy in the presence of the disinfectant. Although bioassays are time-consuming, need purified particles for establishing the quantification models, and are less sensitive than RT-qPCR, they were able to quantify the differences in virus titer in the presence/absence of disinfectant. Interestingly, the bioassays reached at least the lower limit sensitivity of a qELISA. By being less sensitive to the presence of the disinfectant, bioassays proved to be the only technique for the determination of the disinfectant efficacy against ToBRFV on different germ carriers as well as on virus-infected plant sap.

## 1. Introduction

Greenhouses are a part of protected horticulture [1] wherein on the occasion of being contaminated with quarantine or regulated pathogens, proper cleaning and disinfection of all contaminated surfaces have to apply. Viruses are responsible for a significant portion of newly emergent plant diseases [2]. Tomato (*Solanum lycopersicum* L.) is one of the most important vegetables grown in greenhouses worldwide [3]. *Tomato brown rugose fruit virus* (ToBRFV) is a newly emerging virus from the genus *Tobamovirus* in the virus family *Virgaviridae* [4]. The virus is considered a threat to tomato production, especially in greenhouses [5,6,7]. Because of the rapid transmission of tobamoviruses via mechanical contacts, they distribute quickly in the entire greenhouse during hands-on activity or pruning and contaminating different surfaces in the greenhouses, such as tables, pots, and tools [8,9]. Tobamovirus transmission via seeds has been described for some plant species [10]. Tobamoviruses are very stable viruses [11]. It has been shown that the tobacco mosaic virus (TMV) can remain infectious for more than 50 years outside of the host [12,13,14,15,16].

Harnessing this disease is difficult due to the lack of antiviral products applicable to plants. Therefore, the consensus control strategies are consisted of a series of hygienic measures to prevent virus arrival, introduction, and spread into the cultivated plants and cultivation of resistant varieties to minimize crop loss [17]. So, disinfectants play an important role in good hygiene practice for seed treatments, disinfecting tools during hands-on activities, and disinfection of infested areas to prevent virus transmission during the cultivating season and coming cultivation cycles.

Several chemicals have been checked for their potential to be used as a disinfectant that reduces the virus load of plant viruses and viroids in different varieties of plants such as ornamentals [18,19,20], cucurbits [21], and greenhouse tomatoes [22,23,24,25]. Most of these chemicals are not registered and approved as plant protectants in the EU. So far, some studies have been carried out to identify disinfectants against ToBRFV and tobamoviruses for seed treatment [26,27,28,29], soil disinfection [30,31], cloth washing [9], shoes [32], water and nutrition disinfection [33,34,35], tool dipping [22,36,37,38], and inactivation of the virus in plant sap or inoculum [36]. Selection of the most efficient disinfectants and, later, investigation of their impact on the environment is essential. Based on our knowledge, there is no universal method or procedure for the absolute quantification of the efficacy of a disinfectant against plant viruses. Traditionally, to determine the efficacy of disinfectants against fungi and bacteria, the depletion of vital colony-forming units (per mL) on various nutrient media has been evaluated [39,40]. Due to the need for a host cell for virus replication, such a procedure cannot be used for plant pathogenic viruses. Hence, the efficacy of disinfectants against viruses was measured mainly by comparing the number of local lesions or the number of infected plants in the treated sample with regard to the untreated. Therefore, these data are qualitative and cannot be quantitative.

There are several methods for the quantification of plant viruses based on their genome, proteins, pathogenicity, and physical and chemical characteristics. The most common method for the absolute or relative quantification of viruses is the reverse transcription-quantitative polymerase chain reaction (RT-qPCR) [41,42], followed by a quantitative enzyme-linked immunosorbent assay (qELISA) [43,44]. A refinement of quantitative PCR, known as droplet-based digital PCR (ddPCR), can be used for the absolute quantification of plant viruses. In this method, the sample is divided into a number of tiny bioreactors or micro-reaction chambers, and single-molecule amplification is carried out in these bioreactors. The concentration of the target nucleic acid is then calculated using the Poisson distribution by counting the proportion of the fluorescently signal-containing micro-reaction chambers to all the micro-reaction chambers [45]. This method has been recently used for the detection of ToBRFV [46]. Bioassay can also be used for the quantification of plant viruses based on viral pathogenicity. There is a relation between the number of local lesions on the host plants and the titer of a virus in the inoculum [47]. Several mathematical models have been introduced to correlate virus concentration in the suspension and the number of local lesions induced in bioassay [48,49,50,51,52,53,54]. Neither qELISA nor RT-qPCR provides the number of infectious particles in a sample, as only protein or nucleic acid, respectively, is quantified. Although bioassay is less sensitive than molecular methods in the detection of plant viruses, it is a unique method that could verify the infectiosity of a virus.

The choice of the appropriate method should always be based on the investigation aim and the type of sample material. In the case of exclusive detection of a virus, it is a high priority to detect even the lowest concentrations of the virus. A lower virus titer can limit sensitivity by producing false negatives if the virus concentration is under the technique’s detection threshold. Usually, molecular methods are more sensitive than serological ones. Conventional or end-point PCR is much more sensitive than ELISA, and qPCR is more sensitive than the end-point PCR [42,55]. If the disinfectant efficacy is the main objective of a study, the chosen method could detect higher and lower viral load in the starting material and after disinfection, respectively. A complicating factor for all methods is that the quantification of a virus titer is not performed exclusively in suspensions but also on germ carriers made of different materials with different surface structures. The practical tests are oriented toward the future field of application and can take place, for example, on contaminated metal, rubber, textile, or plastic surfaces. These surfaces, as well as the disinfectants to be tested, may hamper correct RNA extraction as the first step for preparation of template subjected to (RT)-PCR and (RT)-qPCR and make this a big challenge. Disinfectants, such as bleach (sodium hypochlorite), hydrogen peroxide, soaps, and the plant protectant MENNO Florades (MF) with its active ingredient benzoic acid, are inhibitors of PCR. They destroy the structure of proteins and break down the nucleic acids in the reactions. Consequently, RT-qPCR might not be applicable in the quantification of the virus load in the presence of these chemicals. Furthermore, the high sensitivity of the amplification techniques could be a disadvantage because contamination of reagents and instruments with amplicons from previous samples and cross-contamination between samples can produce false positives reducing specificity [41].

Since methods are available to quantify viruses based on viral genomes, viral proteins, and pathogenicity, we determined and compared their potential in absolute quantification of the disinfection activity against plant viruses using MENNO Florades and ToBRFV as an example. In this study, we (i) compared the accuracy of qELISA, RT-qPCR, and bioassay for estimating ToBRFV load in an inoculum and (ii) the quantification of the efficacy of a disinfectant applied to suspensions and germ carriers.

## 2. Results

### 2.1. Quantitative Double Antibody Sandwitch—ELISA (qELISA)

**Figure 1:**
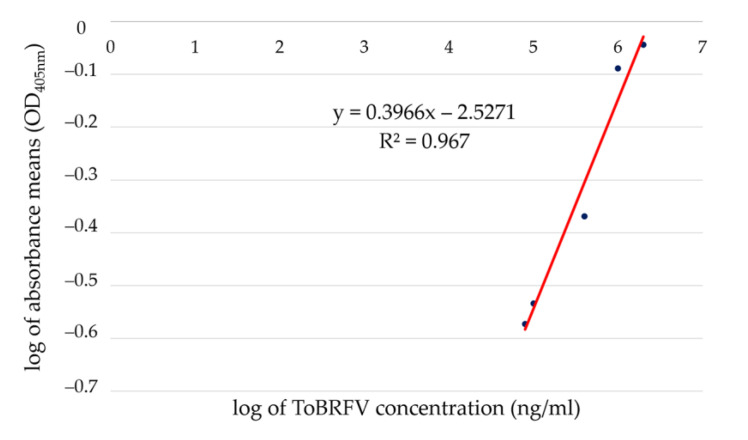
Standard curve for calculating the concentration of target ToBRFV in the samples plotted on log/log scale, 60 min after addition of substrate. Both X and Y axes are on a logarithmic scale. qELISA was carried out with RT-1236 DAS-ELISA kit (DSMZ, Braunschweig, Germany) using a dilution of 1:1000 (*v:v*) and measurement of optical density (OD_405 nm_) 60 min after substrate addition. Each point on the graph represents the mean of the three parallel readings. Y is the log of mean values of the optical density of each concentration or sample, and x is the log of virus concentration in ng/mL.

Quantitative ELISA was used as a protein-based method for the detection and quantification of ToBRFV in different samples. Different concentrations of purified ToBRFV particles were used as references for the establishment of the qELISA standard curve (Section 4.2). The lowest concentration of purified ToBRFV isolate PV-1236 particles detected in qELISA using 1:1000 dilution for antibodies was 0.016 mg/mL (Table 1). Overall, 60 min after the addition of the substrate, there was a linear relationship between the log of optical density mean values and the log of virus concentration in the range of 0.016–2 mg/mL. Based on these data, the equation y = 0.3966x − 2.5271 (R^2^ = 0.967) was used as a reference to determine the viral titers in the analyzed samples (Figure 1 and Table 1). The supernatant obtained from shaking of disinfected and undisinfected (four germ carriers in 2 mL of extraction buffer ELISA in each replication) for 30 min was not detected as positive in qELISA. Elongating the shaking time from 30 min to 3 h did not change the results (Table 1). Based on the qELISA results, the concentration of ToBRFV in the inoculated *Nicotiana benthamiana* and *N. clevelandii* plants were 13.7 mg/mL and 18.5 mg/mL, respectively, in 100 mg of the leaf tissue (Table 1).

### 2.2. Quantification of ToBRFV Concentration via Infectivity (Local Lesion Assay)

Bioassay was chosen as a pathogenicity-based method for the quantification of ToBRFV, and *N. glutinosa* was used as the local lesion host. The number of local lesions after 5–7 dpi (days post inoculation) on the half-leaf units of *N. glutinosa* plants is shown in Table 2. Both the Kleczkowski model [54,56] and the growth curve model [49,54] were fitted to the local lesion number induced by ToBRFV-suspensions with virus concentrations of 128 ng/mL up to 2 mg/mL with the least ${ꭓ}_{i}^{2}$ error, meaning a very good fit (Table 2). Both models fitted best with transformed means of the number of local lesions observed for inoculation of highest and middle concentrations of ToBRFV particles (i.e., 2, 0.016, and 0.0032 mg/mL) (Table 2). However, at the lowest concentration (i.e., 128 ng/mL), there was a slight overestimation of the virus concentration (200–300 ng/mL instead of 128 ng/mL) in our experimental data range, the Kleczkowski model and the growth curve model fitted the data in a both higher and lower concentration of ToBRFV (${ꭓ}_{i}^{2}$ errors: 2.4 and 1.6, respectively). Therefore, equations for the Kleczkowski model [51] and the growth curve model [49] were as follows:

(1) $$Y=\frac{1274.58}{1.81\sqrt{2\pi }\;}\;\int_{-\infty }^{t}\exp\;\left\{-\frac{1}{2}\;{\left(\frac{t-2}{1.81}\right)}^{2}\right\}\;\mathrm{dt}$$

(2) $$Y\left(t\right)=\frac{1134.53}{1+20e^{-1.64t}}$$

These equations were used for the quantification of the virus load [54]. Using the estimated model parameters fitted to the known dilution series, we constructed the inverse function of the Kleczkowski model and the growth curve model, respectively:

(3) $$\log_{10}X=\;t=5.16-\sqrt{2}\;2{\mathrm{erf}}^{-1}\left(-\frac{2Y}{11,064}+1\right)$$

(4) $$X={\left(\frac{1134-Y}{20Y}\right)}^{-1.404}$$

The inverse functions can be used to calculate the expected virus concentration X for a given number of local lesions Y. Both models agree well with each other at the upper and lower ranges of concentrations. Therefore, the concentration of ToBRFV in the inoculated *N. clevelandii* and *N. benthamiana* was estimated at 1.99–2.02 mg/mL and 1.76–1.78 mg/mL, respectively (Table 2).

The inverse functions 3 and 4 revealed that the treatment of plant sap and germ carriers (metal and plastic) containing 1 mg of ToBRFV particles with 4% MENNO Florades for 16 h could reduce the viral load from 1 mg/mL to 4 × 10^−4^, 1.1–1.9 × 10^−4^ and 0–1 × 10^−6^ mg/mL in/on plant sap, plastic and metal germ carries, respectively. As shown in Table 2, MENNO Florades 4% after a 16-h treatment was more efficient in the disinfection of ToBRFV on metal surfaces with a 6-log reduction in the virus load followed by the plant sap and plastic germ carriers with a 4-log- and 3- to 4-log reduction, respectively.

Based on the Kleczkowski model, 0.000321 mg/mL of ToBRFV isolate PV-1236 produces at least one lesion on each of the 24 inoculated half-leaf units. Likewise, 8.37 ng/mL of the virus particles were needed to induce only one lesion in one of the 24 half-leaf units on *N. glutinosa.* Therefore, 8.37 ng/mL is the lowest virus concentration that could be measured in a bioassay test with at least 24 replications.

### 2.3. RNA Extraction

As a first step for the detection and quantification of ToBRFV based on the virus genomic RNA, three different RNA extraction methods, including Spectrum™ Plant Total RNA Kit, RNeasy^®^ Plant Mini Kit, and TRIzol Reagent, were applied. RNA extraction from purified particles, plant sap mixed with ToBRFV particles, and non- and inoculated *N. benthamiana,* and *N. clevelandii* plants resulted in different concentrations of total RNA from the same sample and even between the replications of the same sample ranging from 75 to 650 ng/µL. These three methods were selected to confirm that there was variation in the concentration of extracted RNA from the same source between replications. However, this range was smaller (10–50 ng/µL) when Spectrum™ Plant Total RNA Kit was used. In successful RNA extractions, the minimum RNA integrity number (RIN) of extracted RNA was 7.8. Extracted RNA from 1 mg and 0.1 mg virus particles resulted in 50–87 ng/µL and 10–20 ng/µL (in a total of 50 µL released buffer). However, RNA extraction from 0.01 mg virus particles resulted in a maximum of 5 ng/µL of viral RNA. The RNA extraction of low concentrations of ToBRFV particles was not successful.

In the presence of 4% MENNO Florades or treated samples with MENNO Florades, the maximum concentration of the extracted RNA was 4 ng/µL regardless of the RNA extraction method.

### 2.4. End Point RT-PCR, Cloning, and Sequencing

Endpoint RT-PCR was used to confirm ToBRFV in the inoculated plant. Total RNA extracted from leaves of ToBRFV infected biotest plants were then used to amplify and sequentially clone the nucleotides 1458-6115 of ToBRFV isolate PV-1236 (the numbers are given based on the ToBRFV isolate Tom1-Jo, GenBank accession number: NC-028478) used as template in vitro transcription and then as a standard for the quantification of the viral RNA by RT-qPCR. The expected genomic ToBRFV regions (nucleotides 1458-6115) were successfully amplified via RT-PCR in overlapping fragments using the primer pairs listed in Table 3. These fragments were cloned into pJET1.2 and assembled in a single plasmid/construct. Sequence data confirmed the correct construction of the pJET-ToBRFV-1482-6393 clone.

Endpoint RT-PCR with the primer pairs ToBRFV-1482-s/ToBRFV-1677-as and ToBRFV-2703-s/ToBRFV-2838-as was used to confirm the qELISA results. The expected DNA fragment of 196 bp and 135 bp were documented in inoculated *N. benthamiana* and *N. clevelandii* samples but not in the negative control (non-inoculated *N. benthamiana* and *N. clevelandii* plants). Sequence data confirmed the correct amplification of the partial ToBRFV genome. Therefore, these primer combinations were used for the detection of ToBRFV via endpoint RT-PCR. The optimal thermoprofile for the detection of ToBRFV using these primers was a single step of 50 °C for 30 min and 10 min at 85 °C for cDNA synthesis, and for the PCR, it started with a single step of 2 min at 94 °C, followed by 40 cycles of 30 s at 94 °C, 30 s 50 °C for ToBRFV-1482-s/ToBRFV-1677-as and 60 °C for ToBRFV-2703-s/ ToBRFV-2838-as primer pair, 20 s at 72 °C and a final step of 72 °C for 5 min.

### 2.5. Optimization of RT-qPCR

**Figure 2:**
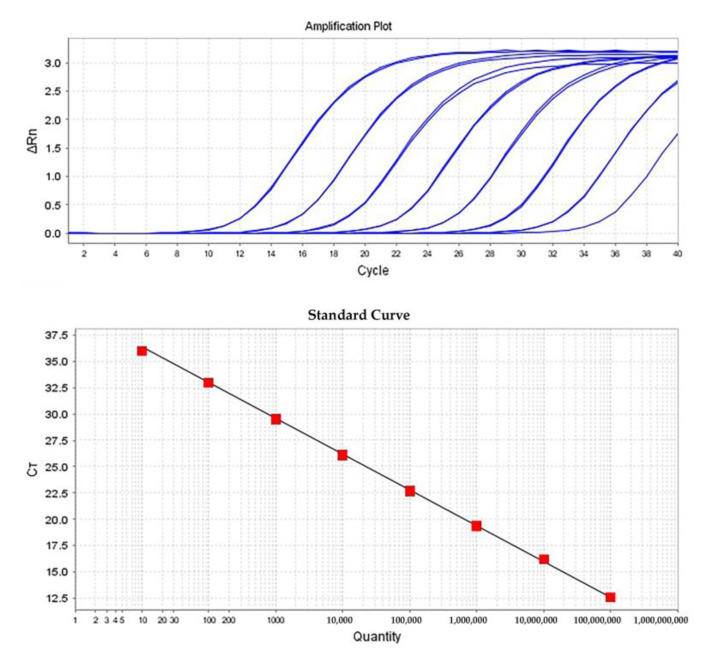
Amplification plot and standard curve of real-time RT-qPCR prepared with 10-fold serial dilutions of in vitro-synthesized RNA transcripts (ToBRFV PV-1236 isolate nucleotide 1482-6393) using real-time RT-qPCR with the primer and probe set ToBRFV-2703-s/ToBRFV-2838-as/ToBRFV-2760 L. Upper graph: amplification curves of a dilution series. The amplification data shown are from triplicate measurements of a 10-fold dilution series with 100,000,000 down to 10 copies per reaction by plotting ∆Rn against cycle. ∆Rn is calculated at each cycle: ∆Rn (cycle) = Rn (cycle)—baseline, where Rn = normalized reporter. Lower graph: standard curves were generated by linear regression analysis, plotting the *Ct* value in the Y-axis versus the logarithm of the starting RNA dilutions in the X-axis. Each plotted point represents the mean *Ct* value that was calculated from the three replicates.

RT-qPCR amplification of the partial ToBRFV RdRP (RNA-dependent RNA polymerase) with the primer and probe set ToBRFV-2703-s/ToBRFV-2838-as/ToBRFV-2760L produced the expected amplicon in the positive samples without any unspecific amplification. There was not any amplification in negative controls. These results were further confirmed by agarose gel electrophoresis and sequencing of the products (data not shown). Amplification curves were sigmoid in shape (Figure 2, upper graph). The RT-qPCR was first optimized by applying different extension temperatures ranging from 55 to 64 °C and then varying the concentrations of primers and probes and different final volumes of the reactions. There were no significant changes in the amplification curves and primer efficiency in the range of 54–62 °C, but the shape of amplification curves was changed at and above 64 °C. Thus, 60 °C was chosen as the extension temperature. There were no changes in the amplification of RT-qPCR when denaturing was performed at 94 °C applied for 1 s to 20 s. Moreover, 0.2 μM each of forward and reverse primers and probe were the optimum concentration, as this gave the highest reporter fluorescence and the lowest *Ct* value.

It could be concluded that RT-PCR and RT-qPCR were not able to detect ToBRFV in the treated samples with MENNO Florades because there was no amplification product, as was also confirmed by the electrophoresis of RT-PCR products. This might be due to a very low amount of extracted RNA.

### 2.6. Quantification of ToBRFV Genomic RNA by RT-qPCR

The standard curves for partial ToBRFV-RdRP, ranging from 1 × 10^8^–10^1^ or 5 copies/µL, were amplified clearly and reproducibly by RT-qPCR (Figure 2 lower graph). The assay was proved to be highly reproducible, as demonstrated by low *Ct* standard deviation values within (three replicates in a single test) and between (each test has been repeated in three individual replication) replicates (Sd: 0.036–0.098) and a high correlation coefficient of the standard curves (*R*^2^ > 0.99). The slope of the standard curve was in the range of −3.332 to −3.436, with a high amplification efficacy of 95.450–99.569%. The *C*t values of the viral target, determined by RT-qPCR for each inoculum used in this study, were inserted into the standard curve formula, y = −3.388x + 39.731 (plot of *Ct* value against the log of the standard sample amount) with the coefficient of determination R² = 0.9988, where y = *Ct* value and x = log copy number (DNA or RNA molecules μL^−1^), allowing to determine the viral titers in the analyzed samples (Table 4). The copy number of the ToBRFV genomic RNA ranged between 3.775 × 10^9^–2.7307 × 10^10^ copy/µg total RNA. The results of the quantification of ToBRFV before and after disinfection based on the qELISA, RT-qPCR, and bioassay are shown in Table 5.

## 3. Discussion

The ToBRFV is a newly emerging virus, representing a major threat to tomato production globally [57]. The virus has been reported in 35 countries in tomatoes (*Solanum lycopersicum* L.), peppers (*Capsicum annuum* L.), or weed hosts [12,58,59]. The ToBRFV has been causing devastating disease outbreaks in tomato production areas [5,6,7,60], causing a yield loss of 15–55% [5]. The virus is subjected to emergency measures in the EU (EU 2020/1191 and further implementations). Exclusion and eradication principles of plant disease management are applied to restrict its entrance and distribution, and propagation materials have to be tested for ToBRFV via RT-PCR and RT-qPCR methods. Moreover, if an infection of ToBRFV is detected in greenhouses, the tomato fruit producers in the EU are obliged to remove and destroy all infected plants from the production site, at the latest, at the end of the cropping season. To prevent the spread of the ToBRFV, producers shall also take special hygienic precautions with regard to personnel, production site buildings, machinery, and pruning instruments (EU 2021/1809).

In the present study, plant sap containing defined virus concentration (a total amount of 1 mg virus particles) has been used and loaded on germ carriers. Hence, the concentration of the initial virus load in all the treated germ carriers and plant sap with the disinfectant was defined. In the literature, generally, propagated virus in a systemic host is used as an inoculum [18,21,24,25,37] in which the concentration of the virus was unknown. In some studies, optical density in ELISA has been used as a tool for the quantification of the virus titer in the initial inoculum [36]. Since there were no standard or reference samples with a defined titer of the virus, the exact amount of the initial virus load in most of the published studies was unknown. In addition, in the lack of a reference or standard sample, any changes in the source of the inoculum and antibody might result in changes in the optical density values, meaning different concentrations of the initial inoculum. These variations could reduce the reproducibility of the results obtained at different times or in different labs. In addition, variations in the virus concentrations have long been observed in different viruses and their host plant species affiliated with different virus variants, plant cultivars, types of tissue, phenological state, or crop stage [61,62,63].

The results of this study showed that the virus concentration in the inocula obtained from *N. benthamiana* or *N. clevelandii* could be different depending on the type of systemic host species. The quantification of the disinfectant efficacy needs the determination of the initial virus load before disinfection treatments and, thereafter, the determination of the remaining virus load. Thus, the chosen method should be functional in both higher and lower concentrations of the virus as well as the presence of the disinfectant. We estimated the accuracy of the three different methods, qELISA, bioassay, and RT-qPCR, in the quantification of the virus load (containing 1 mg/mL virus particles) in the initial inoculum. In the qELISA, the concentration of the initial inoculum was overestimated (1.37 mg/mL instead of 1 mg/mL). In contrast, the concentration of the sample was underestimated in RT-qPCR. Similarly, qELISA quantified ToBRFV concentration in the 100 mg of fresh *N. benthamiana* and *N. clevelandii* leaves as almost 13.7 and 18.5 mg/mL (Table 1), while these concentrations were estimated at almost 1.77 and 2 mg/mL in bioassay and 0.0013 and 0.0023 mg/mL in RT-qPCR for *N. benthamiana* and *N. clevelandii*, respectively. The qELISA and RT-qPCR could also trap/detect incomplete particles and genomic RNAs, which are abundant during virus replication. It has been shown that the coat protein subunits of tobamoviruses form two-layer disks [64], which might interfere with the antibodies and were detected via ELISA. This, in return, might cause an overestimation of actual virus titer in plants. It has been shown that the accuracy of ELISA in quantification also depends on the quality and dilution of the antibodies as well as their attachment to the wells of ELISA plates (binding capacity of ELISA plates). If the ratio of antibody and antigen changes, it directly affects the optical density measurements [65]. Therefore, it is necessary to provide a reference solution for the establishment of the ELISA standard curve. In addition, our results showed that the lowest virus concentration that qELISA could detect (using the DSMZ kit with 1:1000 dilutions of antibodies) was 0.0016 mg/mL. The higher and lower detection limits of ELISA could be increased/decreased by preparation of a higher quality of the antibodies or increasing/reduction in the concentration of applied antibodies in the ELISA reaction in such a way that they do not increase the chance of cross-reactions. The ELISA was also not able to detect the virus particles after treatment with MENNO Florades. The presence of other disinfectants, such as hypochlorite sodium or hydrogen peroxide, which can destroy the protein structure, hamper the detection of the virus.

The RdRP gene was chosen in this study for designing the TaqMan probe and primers for the absolute quantification of ToBRFV because the virus employs a subgenomic RNA strategy to express movement (MP) and coat protein (CP) genes. Therefore, the copy number of the ToBRFV genomic RNA based on the CP and MP genes would be overestimated. The RT-qPCR developed in this study can detect 5 copies of ToBRFV genomic RNA, and RT-qPCR is able to detect less than 10 copies of the virus [66,67,68,69,70,71].

The underestimation of determined virus titer in the spiked samples (samples containing a defined concentration of virus particles or nucleic acid) and in the inoculated plants in RT-qPCR might be because of RNA loss during RNA isolation. RT-qPCR is a powerful and very sensitive method in virus detection and the comparison of virus copy numbers in a defined unit of extracted RNA. Based on our knowledge, there is no report of the quantification of the virus copy number by RT-qPCR in a defined unit of plant tissue. It could be because of technical limitations and challenges, mainly in the RNA extraction procedure, especially in the presence of disinfectants. The results of this study showed that there was no uniformity in the concentration of extracted RNA, not only among the applied methods for RNA extraction but also between the replications of the same sample in the single extraction method. Without a doubt, a portion of RNA was lost during each step of the purification procedure. Moreover, as shown in this study, in the presence of MENNO Florades (4%) in the plant sap, the extraction of RNA had a very low efficacy. It could be assumed that different steps of RNA extraction, such as the efficacy of the extraction buffer, binding of the viral genome to silica columns, or RNA precipitation, could be affected by the chemical properties of the disinfectant. For example, MENNO Florades has a pH = 2, which could change the pH of different solutions. Therefore, the absolute quantification of a virus, i.e., the copy number of viral genomes, is measured only in a defined amount of the final extracted RNA volume, which is in almost all the reported cases based on ng or mg of extracted total RNA. Thus, the estimated copy number or viral genome was not linked to the mg of the starting plant tissue or ml of the virus suspension.

Beside the very low concentration of the extracted RNA in the presence of disinfectant, the isolated RNA can also contain traces of disinfectant. It has been reported that if the template RNA contains chemicals or components that inhibit the PCR (phenols, tannins, and complex polysaccharides), they will affect (RT)-PCR and real-time (RT)-PCR resulting in false negatives. [72,73,74,75,76]. The initial situation is even more complex if the virucidal efficacy of substances has to be tested in virus suspensions, plant homogenates, or germ carriers.

The efficacy of the reverse transcription and PCR depends on the quality of the extracted RNA, the efficacy of the reverse transcriptase enzyme, and the amount of nucleic acid as a template. In addition, the efficacy of the primers plays an important role in the accuracy of the RT-qPCR. It has been shown that the concentration of the RNA in the reverse transcription reaction highly affects the efficacy of this reaction and the synthesis of complementary DNA, cDNA [77]. Another reason for the very low concentration of extracted RNA from dried inoculum on different surfaces was the strong attachment of the inoculum to the surfaces, i.e., plastic and metal. These data show that shaking these materials in a liquid might not be sufficient for releasing or washing the virus particles from these surfaces, and need additional mechanical forces such as brushing to detach the virus from these surfaces. Moreover, it could mean that dried plant sap keeps the virus inoculum on the surfaces. Since tobamoviruses have very stable particles, these contaminated surfaces could serve as a source of inoculum.

Interestingly, with the help of mathematical models, the bioassay used in this study could not only estimate the concentration of initial inoculum before the treatment but also after disinfection. Bioassays do not need RNA extraction; therefore, there is no loss of inoculum. Moreover, it provided sufficient friction as a mechanical force to release dried virus particles from these surfaces. It has been shown that the different models could quantify the ToBRFV titer in the plant sap and virus suspensions [54]. Based on the data presented in Table 2, bioassays could estimate a higher concentration of ToBRFV (e.g., 2 mg/mL), and its lower detection limit was equal to or lower than that of qELISA. The inoculated plants could tolerate the presence of MENNO Florades since there was no phytotoxicity in the inoculated plants. Therefore, the traces of the disinfectant were not affecting the inoculation process.

Some of the disinfectants have phytotoxicity that could also affect the bioassay. It has been shown that 4% MENNO Florades has very light or no phytotoxicity to the plants. It has been shown that a higher concentration than 2 mg/mL of ToBRFV can cause advanced necrosis on the inoculated leaves of *N. glutinosa* [54] due to aggregation of adjacent lesions. MENNO Florades (BVL registration number 044407-00) remains the only authorized plant protectant within the EU to be used for the disinfection of agricultural and horticultural surfaces/objects against harmful plant pathogens. The antimicrobial activity of MENNO Florades is reached by the undissociated molecules of benzoic acid, whose proportion is increased with decreasing pH, and consequently, the effectiveness of the disinfectant is increased in acidic pH conditions [78]. It has been used to control fungi [79], bacteria [79,80], as well as (stable) plant viruses such as ToBRFV [9,32,78]. The data showed that MENNO Florades treatment yielded at least a 4–6 log reduction in the viral load in plant sap and on the metal surfaces and a 3–4 log reduction on plastic surfaces, respectively. In this study, treatment with MENNO Florades for 16 h was used, following the instructions provided by the producer.

In bioassay, the obtained mathematical models showed that 0.000321 mg/mL of the ToBRFV PV-1236 can cause one lesion in all of the inoculated leaves (in all 24 half-leaf units). Inoculation of *N. clevelandii* and *N. benthamiana* with lower concentrations of ToBRFV particles, such as 0.00064 mg/mL and even 0.000128, were infectious with lower frequency of the local lesions in all inoculated leaves. Therefore, it could be concluded that ELISA and bioassay have almost the same lower limit of detection.

It could be concluded that since there is (i) no standard RNA extraction method able to capture all nucleic acids, especially in the presence of disinfectants, (ii) very low reproducibility of the RNA extraction, (iii) no standard method to link the calculated copy number of the viral genome in extracted RNA to the defined amount of the plant tissue (e.g., 1 g of leaf tissue), and (iv) most of the disinfectants are inhibitors of PCR, there is no standard methodology to quantify the efficacy of disinfectants via RT-qPCR. Therefore, our data showed that RT-qPCR is not a good choice for the quantification of virucide efficacy. In this context, qELISA has an advantage over RT-qPCR since there is no RNA extraction method in qELISA. The qELISA can also tolerate the presence of different chemicals in the plant sap, which are usually inhibitors of the RT-PCR and RT-qPCR. However, as shown in this study, there was no exact correlation between the virus concentration and changes in the optical density after the addition of substrate. It has to be emphasized that RT-PCR and RT-qPCR are the most sensitive and reliable methods for the detection of ToBRFV, and based on plant health regulations, RT-PCR and RT-qPCR are recommended for the detection of the virus.

Among three different methods used for the quantification of the disinfectant efficacy, we showed that RT-qPCR and qELISA have some technical problems leading to low efficacy in the presence of the disinfectants, e.g., MENNO Florades. Moreover, because of technical problems in RNA extraction and cDNA synthesis, RT-qPCR cannot be applied at all to quantify the virus load in the initial inoculum, and qELISA overestimated the virus concertation by at least 10-fold. Neither qELISA nor RT-qPCR provides the number of infectious particles in a sample, as only protein or nucleic acid, respectively, is quantified. Although bioassay is a time-consuming method and needs purified particles for establishing the quantification mathematical models, it was the sole method that was able to quantify ToBRFV in the initial inoculum directly and later after the treatment with the disinfectant. In addition, the results of this study showed that the bioassay was as sensitive as qELISA and more tolerant to the presence of disinfectant, making it a unique technique for the quantification of the disinfectant efficacy.

## 4. Materials and Methods

### 4.1. Virus Source

A virus purification (2 mg/mL) of the ToBRFV isolate PV-1236 obtained from DSMZ (DSMZ, Braunschweig, Germany) was used as inoculum. Purified virus particles, as well as ToBRFV-infected *N. benthamiana* and *N. clevelandii* leaves, were used as a source of inoculum in disinfection tests as described before [54]. A local lesion host for ToBRFV, *N. glutinosa,* at the 6–7 well-developed leaf stage was used in bioassay for quantification of the virus.

### 4.2. Quantitative ELISA

Quantitative double antibody sandwich enzyme-linked immunosorbent assay (qELISA) with ToBRFV specific polyclonal antibody (RT-1236, DSMZ, Braunschweig, Germany) was performed following DSMZ instructions for qELISA with 1:1000 dilution of the antibody and conjugate. A 5-fold serially diluted suspension of purified virus particles with a starting concentration of 2 mg/mL was used for the establishment of the standard curve. The 10-fold serially diluted purified virus particles with concentrations of 1, 0.1, and 0.01 mg/mL have also been used to see if these data fit the data. The cut-off value at OD_405_ to identify a ToBRFV-positive sample was calculated based on the following formula: positive and negative cut-off value = negative sample mean + 2 × standard deviations (mean + 2Sd). Microplates with medium binding capacity (Greiner, Kremsmünster, Austria) were used for optimal results. Each sample was tested in triplicate, and the qELISA was repeated at least two times. The plates’ optical density at 405 nm was measured using Multiskan™ FC microplate photometer (Thermo Fisher Scientific, Shanghai, China).

### 4.3. Local Lesion Tests and Models Used for Quantifications Based on the Bioassay

The purified preparation of ToBRFV was used in a dilution series starting from 2 to 0.000128 mg/mL with a dilution factor of 5. The inoculation procedure on the local lesion host *N. glutinosa* is explained elsewhere [54]. However, *N. glutinosa* plants were briefly grown and inoculated when they were in the 5–6 well-developed leaf stages. The plants were inoculated with 100 µL of the inoculum, and the half-leaf units were randomized according to [51] and [54]. Water was used for mock inoculation. The number of the local lesions was recorded at 5–7 dpi (days after inoculation), and the Kleczkowski model [51,56] and growth curve model [49] were used for the quantification of ToBRFV [54].

### 4.4. Disinfection Test

Disinfection efficacy was quantified using different matrices: (i) directly in plant sap and (ii) on germ carriers made of metal or plastic. For the disinfection of plant sap, *N. clevelandii* sap, including 1 mg/mL of purified ToBRFV particles, was mixed with MENNO Florades in a final concentration of 4% and was kept at room temperature for 16–24 h as recommended by the supplier, then 100 µL of this mixture was inoculated on *N. glutinosa* half-leaf units. Metal rings (30 mm in diameter) and plastic pieces (3 × 3 cm) served as germ carriers. An amount of 100 µL of *N. clevelandii* sap harboring ToBRFV particles in a final concentration of 1 mg/mL was loaded on the germ carriers and air-dried by keeping them overnight at room temperature. Subsequently, these carriers were treated with MENNO Florades in a final concentration of 4% and incubated at room temperature for 16–24 h. Instead of MENNO Florades, water was used in control samples. The plant sap and the germ carriers were rubbed on the half-leaf units for inoculation. Mock inoculations, as a negative control, were performed in each case using water. The number of local lesions was monitored 5–7 days post-inoculation (dpi). The concentration of the remaining infectious virus particles in both matrices was calculated based on the standard inoculation curve. Each treatment was applied on 24 half-leaf units (8 × 3) and repeated three times. In addition, germ carriers were dipped in 2 mL extraction buffer in fours and were shaken for at least 30 min while they were immersed in extraction buffer. The obtained extract was subjected to qELISA and RT-qPCR.

### 4.5. RNA Isolation and cDNA Synthesis

Three different methods were used for the extraction of total RNA from purified virus particles, plant, and disinfectant-containing suspensions using (i) Spectrum™ Plant Total RNA Kit (Sigma-Aldrich Catalog No. STRN50, Hilden, Germany) and integrated with DNase I using On-Column DNase I Digest Set (Catalog No. DNASE10 and DNASE70, Hilden, Germany) following the manufacturer’s instructions and final RNA elution with two consecutive washes with 50 μL (final volume of 100 μL) RNase-free water, (ii) RNeasy^®^ Plant Mini Kit (Qiagen, Darmstadt, Germany), and (iii) TRIzol Reagent (Life Technologies, CA, USA) complying with the manufacturer’s instructions. In the third method, a DNase I treatment was applied. A buffer control was included with all of the isolations, as a negative isolation control, for monitoring the potential contamination during the extraction procedures. The concentration of extracted total RNAs was measured with NanoDrop One Microvolume UV-Vis Spectrophotometer (Thermo Fisher Scientific, Waltham, MA, USA). In addition, the quality and quantity of extracted RNA were further checked by a Bioanalyzer RNA 6000 Nano assay (Agilent, Santa Clara, CA, USA), complying with the manufacturer’s recommendations. cDNA synthesis was carried out in a 20 µL reaction volume containing 500 ng of isolated RNA as a template, 20 pmol gene-specific primer, 1X transcriptase buffer, 2 mM dNTPs, 20 units of RNasin, and 100 units of Maxima H minus reverse transcriptase (Thermo Scientific™, Waltham, MA, USA) following the thermal profile recommended by the manufacturer (Thermo Scientific™, Waltham, MA, USA). cDNAs were diluted 1000×–50,000× using DEPC-treated water (Thermo Scientific™, Waltham, MA, USA). A buffer control (no template control), and no reverse transcriptase control were used as negative controls for monitoring the presence of any contamination to the presence of genomic DNA or any other contaminations.

### 4.6. Primers and Probe Design

A nucleotide sequence alignment of the ToBRFV complete genomic sequences available in GenBank before September 2020 was used for designing the primers and probes. Primer Express™ Software v3.0.1 (Applied Biosystems, Waltham, MA, USA) was used for designing the primers. The designed probes and primers were checked for their specificity in silico using BLAST search in NCBI and were synthesized by Biolegio (Nijmegen, The Netherlands), and those used for RT-qPCR were purified by HPLC. TaqMan probe(s) carried a 5′ FAM reporter label and a 3′BHQ1 quencher. Primers used in this study are shown in Table 3.

### 4.7. Cloning and Sequencing of the ToBRFV Partial Genome Used as Standard for RT-qPCR

A total of 1.5 µg total RNA extracted from inoculated *N. clevelandii* was subjected to cDNA synthesis using Maxima H Minus Reverse Transcriptase and random hexamers (Thermo Scientific™, Waltham, MA, USA) following the manufacturer’s protocol. The cDNAs were applied as a template in PCR for amplification of the genomic region of ToBRFV from nucleotides 1482-6393 (numbers are based on the GenBank accession number NC028478) in three segments using the primer pairs of ToBRFV-1482-s/ToBRFV-4750-as, ToBRFV-KpnI-4388-s/ToBRFV-HindIII-6153-as, and ToBRFV-CP-Eco47-s/ToBRFV-HindIII-3UTR-as, and Phusion™ High-Fidelity DNA Polymerase kit (Thermo Scientific, Waltham, MA, USA). PCR products were gel extracted and were inserted into pJET1.2 Blunt end cloning vector (Thermo Scientific, Waltham, MA, USA) in three steps through compatible DNA restriction sites available in the ToBRFV genome, the vector, and the primers. The final recombinant plasmid was named pJET-ToBRFV-1482-6393. The recombinant plasmids were sent to Macrogen (Macrogen Europe, Amsterdam, The Netherlands) for sequencing, and each nucleotide was sequenced at least twice. This plasmid was used as a template for the preparation of the plasmid standard curve in RT-qPCR and in vitro transcription.

### 4.8. Plasmid Standard Curve

The plasmid pJET-ToBRFV-1482-6393 was linearized by *Hind*III restriction enzyme digestion and gel purified. Then the concentration of the linearized plasmid was measured 5 times by UV spectroscopy at 260 nm (NanoDrop™ One/OneC Microvolum-UV/VIS-Spectrophotometer, Thermo Scientific, Waltham, MA, USA), and the mean of the measured values was used for concentration calculation of the plasmid. Then the copy number of the linearized plasmid and the amplicon sequences were calculated using the following equation [81]:

$$\mathrm{DNA}\;\mathrm{copy}\;\mathrm{number}=\frac{6.02\times 10^{23}\;\left(\frac{\mathrm{copy}}{\mathrm{mol}}\right)\times \mathrm{DNA}\;\mathrm{amount}\;\left(g\right)}{\mathrm{DNA}\;\mathrm{length}\;\left(\mathrm{bp}\right)\times 660\;\left(\frac{g}{\mathrm{mol}/\mathrm{bp}}\right)}$$

A serial dilution of a 10-fold factor of the linearized plasmid were prepared to start with 10^7^ copy number.

For correlation of the ToBRFV copy number to the number of virus particles, the molecular weight (MW) of the Tobacco mosaic virus was used because these viruses have almost the same size of genomic RNA (6393–5 nt NC_001367 and NC_028478) and coat protein 17.5 kDa. In addition, the molecular weight of TMV has been calculated as 40 × 10^6^ [82]. Therefore, one mg of the virus consists of 1.505535 × 10^13^ particles. This molecular weight is used for the calculation of ToBRFV particles in this study.

### 4.9. In Vitro Transcription and RNA Standard Curve

Because the T7 promoter implemented almost 50 nucleotides before the insert in pJET-ToBRFV-1482-6393 plasmid, one µg of the HindIII-linearized plasmid was used as template in in vitro transcription reaction, including 10 µL of rNTPs (10 mM each), RiboLock™ RNase Inhibitor (50 u), and T7 RNA Polymerase (30 u) (all Thermo Scientific, Waltham, MA, USA) in a final volume of 50 µL. The reaction was carried out in triplicate and was incubated at 37 °C for 3 h. The template DNA was removed using 2 U of DNase I and incubation at 37 °C for 15 min. The reaction was stopped by the addition of 2 μL 0.5 M EDTA, pH = 8.0, and incubation at 65 °C for 10 min. The in vitro synthesized RNA molecules were recovered by Spectrum™ Plant Total RNA Kit (Catalog No. STRN50, Sigma-Aldrich, St. Louis, MI, USA) following the manufacturer’s instructions for extraction of plant total RNA, and their concentration was measured with Bioanalyzer RNA 6000 Nano assay (Agilent, Santa Clara, CA, USA) complying with the manufacturer’s recommendations. A portion of the purified RNA was used in PCR with specific primers for ToBRFV in triplicate to confirm no DNA template traces. The purified RNA was kept at −80 °C. The copy numbers could be determined by N = C/(K × 330 × 1.6601 × 10^−18^) where N was the copy number per µL, C was the concentration of the sample (µg/µL), K was the length of target gene (nucleotide), and 1.6601 × 10^−18^ was the transfer constant between Dalton and µg.

### 4.10. Absolute RT-qPCR

A primer set of ToBRFV-2703-s/ToBRFV-2838-as with TaqMan probe ToBRFV-2760L was used in RT-qPCR. The RT-qPCR assay in this study was performed using the TaqMan™ Fast Universal PCR Master Mix (2×) kit (Applied Biosystems, Waltham, MA, USA) in reactions of a total of 10 or 20 μL containing 2 or 5 µL diluted cDNAs as a template, respectively. The final concentrations of primers and probes in these reactions were 0.2 pmol of each primer and 0.2 pmol probe, respectively. The amplification condition for the qPCR assay consisted of one cycle of 95 °C for 2 min as a first denaturation and 40 cycles of PCR at 95 °C for 10 s (denaturation) and 60 °C for 20 s (annealing and extension). A buffer control was used for monitoring any contamination in the reactions, and each sample was tested at least in triplicate. TaqMan-based RT-qPCR was performed on the StepOne Real-Time PCR System from Thermo Scientific™ (Waltham, MA, USA). StepOne^TM^ software ver. 2.3 (Life technologies, Carlsbad, CA, USA) was used for analyzing the data. The RT-qPCR products were analyzed further on the 4.5% agarose gel, and sequencing was conducted.

## Figures and Tables

**Table 1:** The measured values of optical density at 405 nm (OD_405 nm_) 60 min after substrate (p-Nitrophenyl phosphate) addition.

Sample Concentration or Name		Log of Virus Concentration	Mean Value of Reads	Log of Mean Values of Reads	Reacted	Calculated Concentration (mg/mL)
Serially diluted ToBRFV particles to establish the standard curve for qELISA	2,000,000 ng/mL	6.3010	0.9045	−0.0436	positive *	1.83
	400,000 ng/mL	5.6021	0.4274	−0.3692	positive	0.47
	80,000 ng/mL	4.9031	0.2674	−0.5728	positive	0.085
	16,000 ng/mL	4.2041	0.2456	−0.6098	positive	0.017
	3200 ng/mL	3.5052	0.1538	−0.8130	negative	NA **
	640 ng/mL	2.8062	0.1031	−0.9867	negative	NA
	128 ng/mL	2.1072	0.1322	−0.8788	negative	NA
	1,000,000 ng/mL	6	0.8156	−0.0885	positive	1.40
	100,000 ng/mL	5	0.2922	−0.5343	positive	0.11
	10,000 ng/mL	4	0.1523	−0.8173	negative	NA
Germ carriers and plant sap loaded by 1 mg ToBRFV particles and treated with 4% MENNO Florades for 16 h	metal carrier untreated with MF		0.0962	−1.0168	negative	NA
	metal carrier disinfected with MF		0.1045	−0.9809	negative	NA
	plastic carrier untreated with MF		0.1012	−1.9208	negative	NA
	plastic carrier disinfected with MF		0.1599	−0.7962	negative	NA
	plant sap untreated with MF		0.0864	−1.0635	negative	NA
	plant sap disinfected with MF		0.0829	−1.0814	negative	NA
Inoculated plants with ToBRFV	*N. benthamiana* plant (1:10 diluted)		0.8069	−0.0932	positive	1.37 (13.7) ***
	*N. clevelandii* plant (1:10 diluted)		0.9091	−0.0414	positive	1.85 (18.52) ***
	Negative control (*N. clevelandii* non-inoculated)		0.1089	−0.9630	negative	NA
	Negative control (*N. benthamiana* non-inoculated)		0.1013	−0.9944	negative	NA
	Substrate (p-Nitrophenyl phosphate)		0.0738	−1.1319	NA	NA

* the calculated cut-off value of ELISA was 0.2338, ** NA not applicable, *** the values in the brackets are final concentration of ToBRFV in 100 mg fresh leaf tissue.

**Table 2:** Mean number of transformed data and model parameters for correlation of the necrotic local lesions on *N. glutinosa* with serially diluted ToBRFV suspensions and computed concentration of ToBRFV in different samples based on the obtained models. Data transformation was performed according to Section 4.3.

	Virus Titer in Inoculum (mg/mL)	$\hat{Y}\;*$	Calculated Virus Load in Inoculum or Remained Virus Load after Treatment with Disinfectant (mg/mL) Based on	
			The Kleczkowski Model [51]	The Growth Curve Model [49]
	2	87.130	2.0629	2.0267
Virus titer in serially diluted ToBRFV isolate PV-1236 particles used as inoculum	0.4	25.909	0.3367	0.3520
	0.08	12.477	0.1167	0.1226
	0.016	2.616	0.0130	0.0128
	0.0032	1.128	0.0042	0.0038
	0.00064	0.493	0.0014	0.0012
	0.000128	0.030	0.0003	0.0002
*N. clevelandii*	unknown	85.9892	2.0218	1.9885
*N. benthamiana*	unknown	41.0477	1.7765	1.7647
disinfected metal carrier **	unknown	0.00689	0.00001	0.00000
disinfected plastic carrier	unknown	0.02001	0.0004	0.00045
disinfected sap	unknown	0.09529	0.00019	0.00011
Model parameters ***			N = 11,064, λ = 2, ξ = 5.16	N= 1134.53, β = 20, γ = 1.64
${ꭓ}_{i}^{2}$			2.40	1.60

* $\hat{Y}$ is the z-equivalent of half-leaf count. First, each local lesion count for a fixed concentration was transformed to normal distribution. $\hat{Y}$ is the inverse transformation of the mean of z-values and is used for fitting the data. $z=\log\;\frac{1}{2}\;[x+c+\surd \left(x^{2}+2\mathrm{cx}\right)$ [56] where c = 20 [54] is used for data transformation. ** each carrier loaded with 1 mg of the virus particles and disinfection was carried out using MENNO Florades 4% for a 16-h treatment, *** N is the mean number of ‘susceptible regions’ per half-leaf which is a hypothetical number, $\xi ={\log\;x}_{0}$ x_0_ = virus concentration or dilution of infective sap when 50% of the susceptible regions develop lesions), and λ is the standard deviation, β and γ are positive constants [49,51,54].

**Table 3:** Characteristics of oligonucleotides used in this study. The position of the nucleotides is shown based on ToBRFV isolate Tom1-Jo (NC-028478).

Name	Sequence 5’ → 3’	Start	End	Application
ToBRFV-1482-s	TAATCAGCAAGTTTAGTTTG	1482	1501	D, C
ToBRFV-1677-as	TCAGTCACTAATCTATCGTG	1677	1658	D, C
ToBRFV-4750-as	GGATCTTCTGAACTCTTCTA	4750	4731	C, I
ToBRFV-4640-s	ATACATCATGACAGAGGGTG	4640	4659	C
ToBRFV-6323-as	GCCTACGGATGTGTATGAAC	6342	6323	C
ToBRFV-KpnI-4388-s	GTTTATGGTACCAGAGAAAGAG	4389	4410	C
ToBRFV-HindIII-6153-as	CTCTAAGCTTACCATTGTAAACCGGATGCAC	6173	6153	C
ToBRFV-CP-Eco47-s	CGTAGAGTAGATGACGCAACG	6050	6070	C
ToBRFV-HindIII-3UTR-as	TATATAAGCTTGCATGCTGGGCCCCTACCGGGGGTTCCGGGGGAAT	6393	6366	C, I
ToBRFV-2703-s	AAGCCACAAGAGATAATGTTCGTA	2703	2726	Q
ToBRFV-2838-as	CAATTTCGCACAGAGACATAG	2858	2838	Q, I
ToBRFV-2760L	6FAM-CTGACAGCGTGTTCCTTTACCG-BHQ1	2773	2752	Q

D, C, I, and Q stand for detection, cloning, in vitro transcription, and absolute RT-qPCR, respectively.

**Table 4:** Overview of estimated ToBRFV load using absolute RT-qPCR with TaqMan probe depended on treatment with the disinfectant MENNO Florades (MF).

Sample	Total RNA Used in cDNA (ng)	*Ct*^1^ ± (SD) ^2^	Quantity ± (SD)	Calculated Virus Concentration		
				Copy No. per µg Total RNA	VP ^3^ (mg) per µg Total RNA	VP (mg) per 100 mg Tissue
0.1 mg virus particles (diluted 1:5000) ^4^	500	17.39 ± (0.087)	2,730,724.75 ± (162233)	2.7307 × 10^10^	0.0018	0.0056
0.5µg in vitro transcript RNA (diluted 1:5000) equal to 0.0126 mg particles ^5^	500	15.264 ± (0.077)	6,738,004 ± (339,407.656)	6.738 × 10^10^	0.0045	NA ^6^
Inoculated *N. clevelandii* (diluted 1:50,000)	500	22.793 ± (0.272)	45,962.41 ± (8171.118)	4.596 × 10^9^	0.0003	0.0023
Inoculated *N. benthamiana* (diluted 1:5000)	500	23.098 ± (0.333)	37,752.36 ± (8331.31)	3.775 × 10^9^	0.0002	0.0013
*N. clevelandii* containing 0.1 mg ToBRFV particles (diluted 1:50,000)	1000	21.644 ± (0.087)	97,568.10 ± (5498.168)	4.88 × 10^9^	0.0003	0.0025
*N. clevelandii* containing 1 mg ToBRFV particles (diluted 1:50,000)(i.e., plant sap untreated with MF)	1000	20.891 ± (0.068)	160,767.54 ± (7322.144)	8.0310^9^	0.0005	0.0128
metal carrier untreated with MF	NA					
metal carrier disinfected with MF	NA					
plastic carrier untreated with MF	NA					
plastic carrier disinfected with MF	NA					
plant sap untreated with MF	NA					

^1^ cycle threshold (Ct) values represent the number of amplification cycles required for a positive PCR result and are a proxy of pathogen quantity in the tested sample, ^2^ standard deviation, ^3^ virus particles, ^4^ 1 mg of ToBRFV particles is equal to 1.5055 × 10^13^ virions or copy number, ^5^ 0.5 µg of in vitro transcribed RNA is equal to 1.063 × 10^11^ particles, ^6^ NA not applicable.

**Table 5:** Overview of estimated ToBRFV load using qELISA, RT-qPCR, and bioassay.

Sample Concentration or Name		qELISA mg/mL	RT-qPCR mg/mL	Bioassay Calculated Virus Concentration (mg/mL)
inoculum	1 mg/mL	1.37	0.0056 ^†^	1 ^‡^
Germ carriers and plant sap loaded by 1 mg ToBRFV particles and treated with 4% MENNO Florades (MF) for 16 h	metal carrier untreated with MF *	NA (negative) **	NA (negative) ***	0.95
	metal carrier disinfected with MF	NA (negative)	NA (negative)	0.000002
	plastic carrier untreated with MF	NA (negative)	NA (negative)	0.93
	plastic carrier disinfected with MF	NA (negative)	NA (negative)	0.00011
	plant sap untreated with MF	NA (negative)	NA (negative)	1.003
	plant sap disinfected with MF	NA (negative)	NA (negative)	0.00011
Inoculated plants with ToBRFV	*N. benthamiana* plant	13.7	0.0013	1.76–1.78
	*N. clevelandii* plant	18.5	0.0023	1.99–2.02

* The metal and plastic germ carriers were loaded with 1 mg of ToBRFV particles. Disinfection was applied using 4% MENNO Florades (MF) for 16 h (Section 4.4), ** NA not applicable, and subsequently, results of the ELISA are given in the brackets, *** the possible reason for negative results might be a very low amount of extracted RNA, ^†^ underestimation of the virus titer in RT-qPCR might be the result of losses of viral particles or virus genome in RNA extraction procedure, ^‡^ the virus titer was calculated by the Equations (3) and (4).

## Data Availability

Not applicable.
